# Potential Vasculoprotective Effects of Blackcurrant (*Ribes nigrum*) Extract in Diabetic KK-A^y^ Mice

**DOI:** 10.3390/molecules26216459

**Published:** 2021-10-26

**Authors:** Kayo Horie, Hayato Maeda, Naoki Nanashima, Indrawati Oey

**Affiliations:** 1 Department of Bioscience and Laboratory Medicine, Hirosaki University Graduate School of Health Sciences, Hirosaki 036-8564, Japan; nnaoki@hirosaki-u.ac.jp; 2 Faculty of Agriculture and Life Science, Hirosaki University, Hirosaki 036-8561, Japan; hayatosp@hirosaki-u.ac.jp; 3 Department of Food Science, University of Otago, P.O. Box 56, Dunedin 9054, New Zealand; indrawati.oey@otago.ac.nz; 4 Riddet Institute, Massey University, Private Bag 11 222, Palmerston North 4442, New Zealand

**Keywords:** polyphenol, blackcurrant extract, diabetes model animal, endothelial nitric oxide (eNOS), alpha-smooth muscle actin (α-SMA)

## Abstract

Polyphenols are bioactive compounds found naturally in fruits and vegetables; they are widely used in disease prevention and health maintenance. Polyphenol-rich blackcurrant extract (BCE) exerts beneficial effects on vascular health in menopausal model animals. However, the vasculoprotective effects in diabetes mellitus (DM) and atherosclerotic vascular disease secondary to DM are unknown. Therefore, we investigated whether BCE is effective in preventing atherosclerosis using KK-A^y^ mice as a diabetes model. The mice were divided into three groups and fed a high-fat diet supplemented with 1% BCE (BCE1), 3% BCE (BCE2), or Control for 9 weeks. The mice in the BCE2 group showed a considerable reduction in the disturbance of elastic lamina, foam cell formation, and vascular remodeling compared to those in the BCE1 and Control groups. Immunohistochemical staining indicated that the score of endothelial nitric oxide synthase staining intensity was significantly higher in both BCE2 (2.9) and BCE1 (1.9) compared to that in the Control (1.1). Furthermore, the score for the percentage of alpha-smooth muscle actin was significantly lower in the BCE2 (2.9%) than in the Control (2.1%). Our results suggest that the intake of anthocyanin-rich BCE could have beneficial effects on the blood vessels of diabetic patients.

## 1. Introduction

Diabetes mellitus (DM) is a serious metabolic disorder with an increasing incidence worldwide. According to the International Diabetes Federation, 1 in 11 adults has diabetes, 90% of whom have type 2 DM [1,2]. Cardiovascular disease (CVD) caused by atherosclerosis secondary to DM has been proposed as an important risk factor for death in patients with diabetes, as it significantly reduces the quality of life of the patients [3,4,5,6,7,8]. Furthermore, CVD caused by atherosclerosis in patients with diabetes has been reported to be more severe than that in patients without DM [9,10]. Therefore, prevention of atherosclerosis is crucial for patients with DM.

The adoption of a recommended diet is recognized as promoting human health, and dietary modification significantly reduces the risk of diseases [11,12].

Polyphenols are biologically active compounds naturally derived from fruits and vegetables and are widely used in disease prevention and health maintenance. In addition, many studies have supported the beneficial effects of polyphenols in CVD or diabetes [13,14,15,16]. Blackcurrants (*Ribes nigrum*) contain four major anthocyanins: cyanidin-3-glucoside, cyanidin-3-rutinoside, delphinidin-3-glucoside, and delphinidin-3-rutinoside. Cyanidin-3-rutinoside and delphinidin-3-rutinoside are the major components in blackcurrants [17]. Blackcurrant extract (BCE) has several health benefits: it improves glucose metabolism [18,19,20,21] and dyslipidemia in high-fat, diet-fed animals [22,23] and promotes cardiovascular health in humans [24,25,26]. We previously reported the direct effects of BCE in preventing vascular disorders [27]; however, to the best of our knowledge, there are no other studies on this topic.

In our previous studies on ovariectomized (OVX) rats, which are widely used as animal models of menopause, BCE administration exerted beneficial effects on vascular health. These included an increase in the level of endothelial nitric oxide synthase (eNOS), which is beneficial for improving vascular endothelial function [28] and in the prevention of elastin degradation and vascular remodeling [27]. Moreover, we found that BCE effectively reduced lipid metabolism abnormalities, suggesting its use as an effective strategy in preventing atherosclerosis [29]. The daily intake of BCE exerts a hypocholesterolemic effect in healthy young women [30], and it can attenuate smoking-induced acute endothelial dysfunction and improve peripheral temperatures in young smokers [31]. These results suggest that the intake of BCE is effective in preventing atherosclerosis, not only in the context of menopause as observed in OVX rats, but also in men and women of all ages. To the best of our knowledge, this is the first histopathological study examining the beneficial effects of BCE on vascular health in diabetes.

Vascular remodeling likely occurs in diabetes and contributes to the development of complications [32]. Furthermore, hyperglycemia is one of the main factors involved in the pathogenesis of atherosclerosis, and, in diabetes, this remodeling extends to the capillaries, microvascular beds, and arteries to different degrees [33]. We previously demonstrated that BCE treatment exerted beneficial effects on vascular health in OVX rats. Specifically, we observed pathological vascular remodeling only in the OVX rats that did not receive BCE treatment; none of the rats treated with 3% BCE exhibited remodeling. In addition, the 3% BCE-treated rats showed reduced elastin fragmentation compared to those in the Control group [27]. Stary et al. [34] provided a histological classification of atherosclerosis according to three characteristic lesion types (Types I–III). Type I lesions represent the initial changes characterized by an increase in the number of intimal macrophages and the appearance of macrophages filled with lipid droplets (foam cells). Furthermore, endothelial dysfunction is regarded as a hallmark of diverse pan-vascular diseases in humans, including atherosclerosis, hypertension, and diabetes [35], and is well known to initiate atherosclerosis [36,37,38]. In addition, eNOS-derived nitric oxide (NO) release plays an important role in secondary atherosclerosis in diabetes [39,40,41].

Furthermore, vascular smooth muscle cells (VSMCs) are critical for maintaining the integrity of the arterial wall; they participate in vascular remodeling and play an important role in atherosclerosis throughout disease progression [42]. Therefore, it is also necessary to evaluate the expression levels of eNOS and alpha-smooth muscle actin (α-SMA) proteins in the blood vessels. The KK-A^y^ mouse represents a combined model created by the introduction of the A^y^ gene into KK mice, which was established by Nishimura et al. in 1969 [43]. These mice exhibit obesity and hyperglycemia, including high levels of HbA1c and albuminuria, and thus are widely used as an animal model for type 2 diabetes.

We assessed the effects of two doses of BCE (1% and 3%) on KK-A^y^ mice using histological parameters to ascertain whether BCE can ameliorate diabetes-associated atherosclerosis. The aim of the present study was to clarify the beneficial effects of polyphenol-rich BCE on vascular health in diabetes.

## 2. Results and Discussion

### 2.1. Histological Analyses of the Abdominal Aorta of BCE-Treated KK-A^y^ Mice

**Figure 1:**
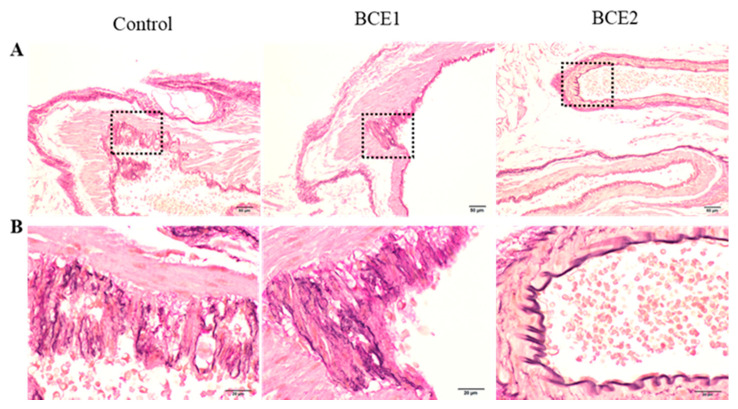
Representative images of foam cell uptake in the tunica intima obtained using Elastica van Gieson staining in the Control (high-fat diet without BCE), BCE1 (high-fat diet supplemented with 1% BCE powder), and BCE2 (high-fat diet supplemented with 3% BCE powder) groups. (**A**) Lower magnification image (100×, scale bar = 50 µm) and (**B**) higher magnification image of the boxed areas shown in panel A (magnification 400×, scale bar = 20 µm).

We assessed the effects of BCE in the aorta of KK-A^y^ mice using Elastica van Gieson staining (Figure 1). The high-fat diet thickened the tunica media as a whole. Although structural irregularities were observed in the blood vessels of all mice in the Control and BCE1 groups, some specimens of the BCE2 groups appeared to have an almost normal vessel structure. In addition, the elastin fibers of the internal elastic lamina were disturbed in the majority of the Control and BCE1 groups, but they were relatively less disturbed in BCE2 groups. Some mice in the BCE2 group even showed a normal internal elastic lamina with a regular structure, which was not observed in any of the mice from the other two groups.

Microscopic observation demonstrated the presence of foam cells in the tunica intima of the aortas of both Control and BCE1 groups. These cells were brightly stained, indicating the uptake of lipid droplets in the cytoplasm of macrophages. However, no obvious foam cells were observed in the BCE2 group.

The relationship between arteriosclerosis and intimal macrophages in KK-A^y^ mice has been reported. Mita et al. reported that the arteries of KK-A^y^ mice fed a high-cholesterol diet show enhanced adhesion of monocytes to the endothelial cells along with the development of atherosclerotic lesions [44]. The ABC transporter ABCG1 plays a major role in foam cell formation in type 2 diabetic KK-A^y^ mice; therefore, it is an important target for the prevention of atherosclerosis [45]. The intake of a high fat diet in KK-A^y^ mice could be the cause of the appearance of foam cells.

To date, many studies have reported that polyphenol-rich foods have hypocholesterolemic effects and cardiovascular benefits [22,23,46,47,48,49,50,51]. Although we did not examine KK-A^y^ mice’s serum lipids in this study, we previously investigated the serum levels of total cholesterol, low-density lipoprotein (LDL) cholesterol, and triglycerides in OVX rats fed a 3-month diet either supplemented with 3% BCE or not supplemented as a Control. The lipid levels were significantly lower in the BCE-supplemented group, demonstrating that BCE intake improves lipid metabolism [29], in agreement with the results of other studies [22,23]. Furthermore, we performed another study to verify the effects of BCE in humans, in which 12 young healthy women were administered BCE for approximately 1 month. The results showed that the levels of total cholesterol and very-low-density lipoprotein cholesterol decreased significantly, and the LDL cholesterol level showed a decreasing tendency [30]. Therefore, administration of BCE has the potential to reduce lipid metabolism abnormalities.

In the present study, supplementation of a high-fat diet with 3% BCE apparently protected against disturbance of the elastic lamina, the generation of foam cells, and vascular remodeling, suggesting that BCE can also ameliorate dyslipidemia based on our previous results. However, our specimens did not exhibit any characteristics that fit the definition of advanced atherosclerosis [52], such as fatty streaks, fibrous plaque, arterial wall thickening, and vascular lumen stenosis. In the present study, the KK-A^y^ mice were evaluated after being administered BCE for 9 weeks. In our previous studies, we demonstrated beneficial effects on vascular health and lipid metabolism in OVX rats fed BCE for 3 months [27,28,29]. Hence, there is a possibility that the preventive effects of BCE on atherosclerosis secondary to diabetes may have been observed if this study was conducted over a longer period.

### 2.2. eNOS Protein Expression in BCE-Treated KK-A^y^ Mice

**Figure 2:**
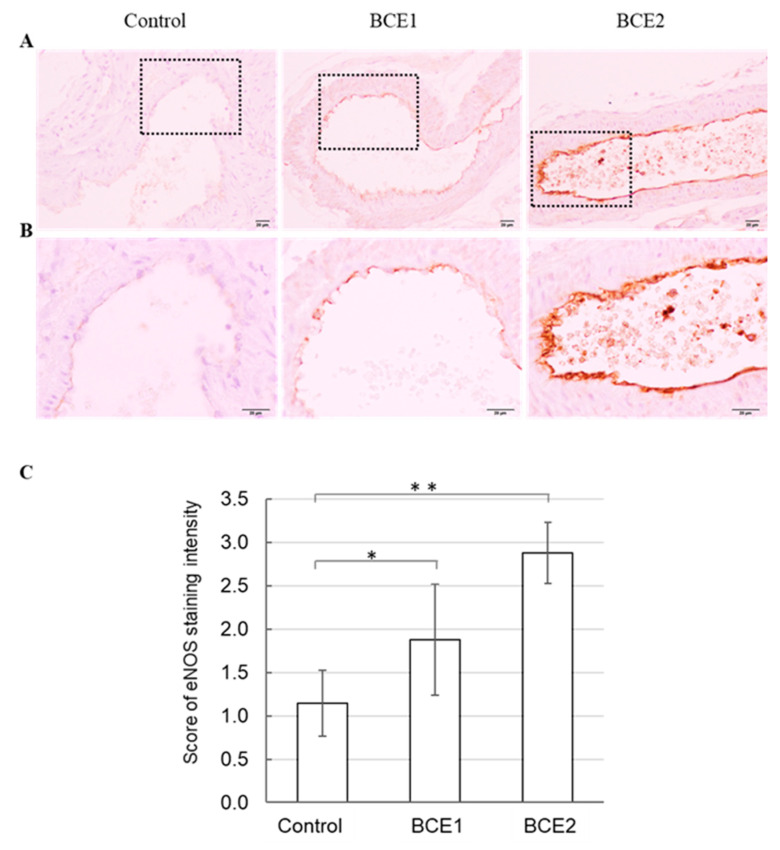
Immunohistochemical staining of eNOS in the Control (high-fat diet without BCE), BCE1 (high-fat diet supplemented with 1% BCE powder), and BCE2 (high-fat diet supplemented with 3% BCE powder) groups. (**A**) eNOS expression at a lower magnification (200×, scale bar = 20 µm). (**B**) A higher magnification image of the boxed areas shown in panel A (400×, scale bar = 20 µm). (**C**) Scoring of staining intensity of eNOS. Data are expressed as the mean score ± standard error. * *p* < 0.05, ** *p* < 0.01.

As eNOS plays a central role in maintaining endothelial function [53], we assessed whether the intake of BCE increased the expression of eNOS by immunohistochemical staining of the aortas of the KK-A^y^ mice from the three groups (Figure 2A,B). eNOS was expressed in the cytoplasm of the endothelial cells, with significantly higher expression in the BCE2 and BCE1 groups than in the Control group. In addition, eNOS expression was higher in the BCE2 group than in the BCE1 group (Figure 2C).

Nitric oxide (NO) is a pivotal vasoprotective molecule that is released from endothelial cells via eNOS activity [54,55]. Furthermore, polyphenols have been shown to influence NO synthesis by regulating eNOS expression in animal disease models. Taguchi et al. [56] reported that the plant polyphenols morin and quercetin promote eNOS-mediated NO production and vasodilation in the aortas of streptozotocin-induced diabetic mice. Huang et al. [57] reported that green tea polyphenols promote reendothelialization in diabetic rabbits by reactivating the Akt/eNOS pathway. Furuuchi et al. [58] showed that boysenberry polyphenol and anthocyanins (the main component of boysenberry polyphenol) increase NO production in a diet-induced obese mouse model. According to our previous study, both anthocyanins and BCE strongly increase NO production and eNOS mRNA expression in human endothelial cells, and dietary BCE markedly increases the eNOS levels in the blood vessels of OVX rats [28]. Consistent with these results, the present study also showed that the dietary intake of polyphenol-rich BCE increased the eNOS level in the blood vessels of KK-A^y^ mice. Hence, it can be hypothesized that BCE plays a role in eNOS activation, which may lead to the prevention of secondary atherosclerosis in diabetes.

### 2.3. α-SMA Expression in BCE-Treated KK-A^y^ Mice

**Figure 3:**
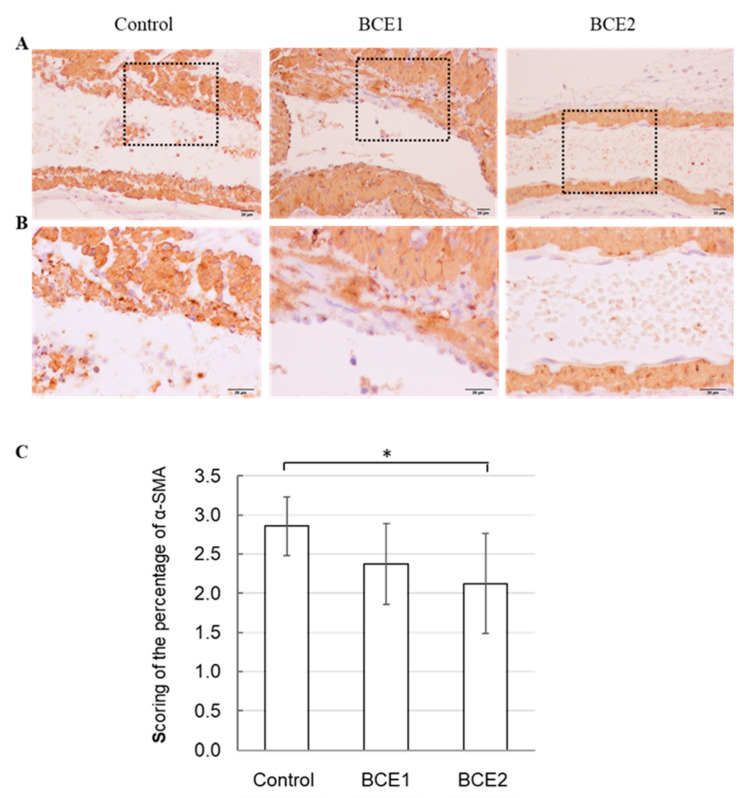
Immunohistochemical staining of α-SMA in the Control (high-fat diet without BCE), BCE1 (high-fat diet supplemented with 1% BCE powder), and BCE2 (high-fat diet supplemented with 3% BCE powder) groups. (**A**) α-SMA expression at a lower magnification (200×, scale bar = 20 µm). (**B**) A higher magnification of the boxed areas shown in panel A (400×, scale bar = 20 µm). (**C**) Scoring of the percentage of α-SMA-positive cells in the tunica intima of the specimens from each group. Data are expressed as the mean score ± standard error. * *p* < 0.05.

Furthermore, in vitro mechanistic studies and in vivo correlative data suggest that VSMCs play an important role in the initiation of atherosclerosis [59]. α-SMA is the actin isoform that is predominant in VSMCs [60]. Thus, we used α-SMA as a marker for VSMCs with immunohistochemical staining and investigated the percentage of α-SMA-positive cells in the tunica intima. The results showed that the migration of VSMCs occurred in almost all specimens examined from the three groups. However, the score of the percentage of α-SMA-positive cells was significantly lower in the BCE2 group than in the Control group, and there was a tendency for a lower positivity rate than that in the BCE1 group (Figure 3C).

Our previous study also showed reduced expression of α-SMA in BCE-treated OVX rats compared with that in the Control rats [27]. In addition, Lin et al. [61] reported that the plant polyphenol compound pterostilbene, which is mainly found in blueberries, may inhibit smooth muscle cell migration. Thus, the results of the present study suggest that the intake of polyphenol-rich BCE may inhibit VSMC migration in KK-A^y^ mice, which might in turn be associated with the prevention of vascular remodeling.

In this study, we focused on the inhibitory effect of BCE on arteriosclerosis secondary to diabetes; therefore, we did not evaluate the direct anti-diabetic effect of BCE. However, BCE is a traditional medicine known for its use in the management of diabetes [62]. In addition, BCE could exhibit anti-diabetic activity, as ascertained from earlier studies. Dietary BCE significantly reduces blood glucose concentration in KK-A^y^ mice [19]. In addition, the α-amylase and α-glucosidase inhibitory activities increased when BCE was added to oat bran paste, suggesting that BCE could be a bioactive component with antidiabetic activity [63].

## 3. Materials and Methods

### 3.1. BCE and BCE-Containing Feed

The powder form of BCE (CaNZac-35) was purchased from Koyo Mercantile Co., Ltd. (Tokyo, Japan). Briefly, the squeezed juice residue was freeze dried and then extracted with ethanol; the extract was concentrated by passing through a filter and the filtrate was freeze dried again. This powder contained a high concentration of anthocyanins (38.0 g/100 g BCE powder) [64]. The BCE-containing feed was prepared as described previously [27] by supplementing AIN-93G-based high-fat diet (CLEA Japan, Inc., Tokyo, Japan) (20% fat) with 1% or 3% BCE powder.

### 3.2. Animals and Treatments

This study was conducted in accordance with the Guidelines of Hirosaki University for Animal Experimentation (permission number: A 17002). Female KK-A^y^ mice (3 years old) were purchased from CLEA Japan, Inc., Tokyo, Japan. The animals were housed in an air-controlled room (temperature, 23 ± 1 °C) with a 12/12 h light/dark cycle. The mice were provided ad libitum access to standard diet and tap water. They were individually housed in plastic cages. After acclimatization for 1 week, the mice were fed the AIN-93G-based high-fat diet and divided into the following three groups: the Control group without BCE supplementation (*n* = 9), the BCE1 group with dietary supplementation of 1% BCE powder (*n* = 8), and the BCE2 group with dietary supplementation of 3% BCE powder (*n* = 8). The BCE concentrations were decided on the basis of preliminary tests in mice, which were designed based on the results obtained for cell lines. The experimental diets were fed to the mice for 9 weeks. The food intake was measured during the experiment. At the end of the experiment, the animals were euthanized and the abdominal aorta was collected for evaluation.

### 3.3. Histological Analyses

The aorta tissue samples were fixed in 10% formalin neutral buffer solution and routinely processed for paraffin embedding. Serial 3-μm-thick sections were placed on glass slides and subjected to Elastica van Gieson staining (Muto Pure Chemicals, Tokyo, Japan) for histological analysis. The stained specimens were photographed using an AX80 DP21 digital microscope camera (Olympus, Tokyo, Japan) interfaced with a computer and evaluated as previously described [27].

### 3.4. Immunohistochemical Staining of eNOS and α-SMA

Before immunohistochemical staining, we performed an antigen retrieval step by boiling the specimens in ethylenediaminetetraacetic acid (EDTA) buffer (pH = 8.5) (Nippon Gene, Tokyo, Japan) using a microwave oven for 20 min. Endogenous peroxidases in the specimens were blocked using peroxidase-blocking solution (DakoCytomation A/S, Glostrup, Denmark) for 5 min at room temperature, followed by incubation with Protein Block Serum-Free reagent (DakoCytomation A/S, Glostrup, Denmark) for 5 min at room temperature. The sections were incubated with the following primary antibodies: anti-eNOS rabbit polyclonal antibody (prediluted; Abcam, Tokyo, Japan) or anti-ACTA2/α-SMA rabbit polyclonal antibody (1:100; Proteintech Group, Chicago, IL, USA) at room temperature for 90 min, and then washed. The tissue sections were incubated with EnVision™/HRP Rabbit/Mouse secondary antibodies (DakoCytomation A/S) for 30 min at room temperature and visualized using the chromogen 3,3-diaminobenzidine. Nuclei were counterstained using Mayer’s hematoxylin (Wako Pure Chemical Industries, Ltd., Osaka, Japan) for 5 min at room temperature. Immunohistochemical staining was visualized using the EnVision™ detection system (DakoCytomation A/S) following the manufacturer’s recommendations. The scores of eNOS staining intensity were determined semi-quantitatively, as described previously (weak—1; moderate—2; intense—3) [28]. The scores of α-SMA were determined semi-quantitatively as the percentage of α-SMA-positive cells in the tunica intima in the blood vessel from each specimen, as follows: <30%—1; ≥30% but <80%—2; ≥80%—3.

### 3.5. Statistical Analysis

Results are expressed as mean ± standard error of the mean. All statistical tests were performed using BellCurve for Excel ver. 3.10 (Social Survey Research Information, Tokyo, Japan) and Kruskal–Wallis analysis with Steel–Dwass post hoc test; *p* < 0.05 was considered to indicate statistical significance.

## 4. Conclusions

Anthocyanin-rich BCE has beneficial effects on vascular health and reduces the degree of lipid abnormalities via phytoestrogenic activity in an animal model of menopause; however, it was unclear whether BCE has similar effects on diabetes. This study indicated that the dietary intake of BCE led to significantly higher expression of eNOS in KK-A^y^ diabetic mice. Furthermore, BCE prevented vascular remodeling and elastic lamina disruption, reduced foam cells, and inhibited VSMC migration. Taken together, these results suggest that the intake of anthocyanin-rich BCE could confer beneficial health effects by preserving the blood vessels of patients with diabetes. In this study, we used aged female mice considering the fact that the risk of cardiovascular disease increases in menopausal woman. In the future, it will be necessary to conduct similar experiments using aged male mice. In addition, we performed only histological evaluations, and therefore, future studies evaluating other parameters or elucidating the underlying mechanisms are needed. Maintaining vascular integrity is critical for preventing secondary arteriosclerosis in diabetes, and we intend to perform clinical studies to validate these results.

## Data Availability

The data presented in this study are available in this article.

## Sample Availability

Samples of the BCE powder are available from the authors.
